# The Use of Older Versus Newer Data in the National Surgical Quality Improvement Program Database Influences the Results of Total Hip Arthroplasty Outcomes Studies

**DOI:** 10.5435/JAAOSGlobal-D-19-00108

**Published:** 2019-10-02

**Authors:** Blake N. Shultz, Anoop R. Galivanche, Taylor D. Ottesen, Patawut Bovonratwet, Jonathan N. Grauer

**Affiliations:** From the Department of Orthopaedics and Rehabilitation, Yale School of Medicine, New Haven, CT.

## Abstract

**Introduction:**

National databases, such as the National Surgical Quality Improvement Program (NSQIP) database, are frequently used for total hip arthroplasty (THA) studies. NSQIP variables and the population included in the database have evolved over time. These changes may influence the results of studies using different periods of data.

**Methods:**

THA patients were aggregated from the 2005 to 2010 and 2011 to 2015 NSQIP data sets to define two era groups. Demographic data and 30-day perioperative outcomes were compared between the groups. As an example analysis, multivariate Poisson regression was used to determine the correlation between age and perioperative outcomes for each group.

**Results:**

Of 102,411 THA patients identified, 8098 cases were from 2005 to 2010 and 94,313 were from 2011 to 2015. A number of preoperative characteristics and perioperative outcomes were significantly different between the era groups. Multivariate analysis of the 2005 to 2010 group showed that increasing age was significantly associated with urinary tract infection and length of stay (LOS), and multivariate analysis of the 2011 to 2015 group showed that age was significantly associated with urinary tract infection, LOS, 30-day mortality, unplanned reintubation, extended LOS, pneumonia, deep vein thrombosis/thrombophlebitis, blood transfusion, and return to the operating room.

**Conclusion:**

Significantly more THA patients were enrolled in the NSQIP in the years between 2005 and 2015. Populations in 2005 to 2010 versus 2011 to 2015 were associated with differences in preoperative characteristics and perioperative outcomes. In an example analysis, it was shown that these differences together lead to different study results and conclusions. This needs to be considered when interpreting and conducting studies using earlier NSQIP data.

Data from national databases such as the American College of Surgeons' National Surgical Quality Improvement Program (NSQIP) database are commonly used to study outcomes after an orthopaedic surgery. In fact, in the last year alone, nine studies have been published on total hip arthroplasty (THA) using NSQIP data.^1,2,3,4,5,6,7,8,9^

The number of hospitals participating in NSQIP data collection, as well as the representative portion of cases captured at many hospitals, has increased since its inception in 2005. This has resulted in an ever-growing population of patients in the database. In the 2005 to 2006 data set, a total of 152,490 cases were included from 121 hospitals. This number swelled to 885,502 cases from 603 participating sites in 2015.^10^ The growth of this database has allowed for greater numbers of patients to be studied after procedures such as THA. For example, a study using data from 2005 to 2007 was able to examine only 584 THA patients,^11^ whereas a later study using data from 2010 to 2013 was able to study 39,334 THA patients.^8^

Another set of differences in the NSQIP over the years has been the variables collected and the definitions of some of those variables. To that end, a total of 274 variables were collected in 2005, peaking at 323 variables in 2014, and subsequently reduced to 273 in 2015.^10^ Examples of the variables included in early years which are no longer collected include a history of myocardial infarction and a history of transient ischemic attack. Furthermore, a number of variables used in common comorbidity indices including the Charlson Comorbidity Index and the modified Frailty Index are no longer collected, diminishing their utility in longitudinal studies and preventing their use in recent years.^12,13^ Variables that have been added over the years include *International Classification of Diseases, Tenth Revision* codes indicating reasons for readmission. Variables that have changed in definition over the years include diabetes, hypertension, and perioperative blood transfusion. Although these changes may be necessary to reflect changes in clinical knowledge, they make interpreting longitudinal studies in the NSQIP challenging for those without deep knowledge of the database.

Although previous studies have suggested that the results of identical outcomes studies can vary between different databases, few studies have looked at how changes within a single database can influence the results across the years.^14,15,16,17^ Consideration of these changes is particularly important in the case of THA, as a number of recent articles have used only older data (before 2011). Of note, at least eight studies from the last 4 years used only data before 2011.^11,18,19,20,21,22,23,24^

A previous study of lumbar fusion patients suggested that systemic changes within the NSQIP can alter the results of common analyses using data from the NSQIP years before 2011 versus more recent years.^25^ Therefore, this current study aims to consider the differences in NSQIP THA patient populations over the years and the potential effect of such differences on study outcomes. If the hypothesis is true that focusing on earlier years of the database affects the study results, readers should exercise caution when interpreting and using the results of studies using only early data. Furthermore, repetition of these studies using more recent data may be appropriate.

## Methods

### Data Source and Study Population

The NSQIP database was used to conduct a retrospective cohort study of prospectively collected data. This study received exemption from our institution's Human Investigations Committee. The NSQIP is a large, national database administered by the American College of Surgeons which uses onsite clinical reviewers at participating hospitals to prospectively identify patients and collect data from surgical reports, medical records, and patient interviews. Data are collected for 30 days after the index procedure, regardless of discharge status. The data are validated using rigorous internal audits which have found a disagreement rate of less than 2%.^10^

Patients who underwent THA were identified from the 2005 to 2015 NSQIP databases using the primary *Current Procedural Terminology code* 27130. Then, cases performed for tumors, fractures, and infections were excluded along with emergent cases. Finally, patients with missing data for any of the studied perioperative characteristics that were collected in a given year were excluded.

To compare the results generated through analyses of older versus newer data, two era groups were created. As described in Shultz et al,^25^ a previous study in lumbar fusion patients, the greatest number of data definition changes occurred in 2011 (see Appendices A and B, http://links.lww.com/JG9/A58 and http://links.lww.com/JG9/A59). In 2011, new, smaller clinical centers were allowed to collect a reduced set of variables. Collection of these variables was ceased at all centers in 2013.^26^ Therefore, data from 2005 to 2010 were grouped together as older years and those from 2011 to 2015 were grouped as more recent years. This division also created an equal number of data set years within each group. The collection patterns of the variables studied were analyzed, and variables no longer collected beginning in 2011 were indicated where appropriate.

### Population Characteristics

Demographic variables abstracted from the database included age, race, sex, height, and weight. Body mass index was calculated from height and weight, and morbid obesity was defined as a body mass index greater than 40 kg/m^2^.

Comorbidity variables directly abstracted from the database included smoking history, alcohol use, American Society of Anesthesiologists (ASA) class, diabetes (insulin dependent and non–insulin dependent), hypertension, congestive heart failure, steroid use, bleeding disorder, and functional status.

Other categorical variables were created to capture multiple NSQIP variables, as defined in a previous study.^25^ Pulmonary comorbidity was defined as any requirement for ventilator-assisted respiration in the 48 hours before surgery, pneumonia, and/or a history of chronic obstructive pulmonary disease. Cardiac comorbidity was indicated for patients with congestive heart failure, previous percutaneous coronary intervention, myocardial infarction, a history of angina, and/or previous cardiovascular surgery of another type. Renal comorbidity was indicated by either dialysis before surgery or acute renal failure. Neurologic comorbidity included hemiplegia, impaired sensorium, a history of transient ischemic attacks, quadriplegia, paraplegia, coma of >24 hours, and/or cerebrovascular accident with or without neurologic deficit.

Surgical time (in minutes), hospital length of stay (LOS) (in days), and perioperative outcome variables were also directly extracted from the NSQIP database. Extended LOS was defined as an LOS greater than or equal to 1 SD above the mean LOS.

### Comparison of Populations Between the Era Groups

All statistical analyses were conducted using Stata version 13.0 (StataCorp, LP). Bivariate analysis was used to compare demographic data and perioperative outcomes to highlight potential differences between the 2005 to 2010 and 2011 to 2015 era groups. Pearson chi-squared test was used for categorical variables, one-way analysis of variance for continuous variables, and the Kruskal-Wallis test for ordinal variables (ASA class). A Bonferroni correction was used to correct for the multiple tests carried out, yielding a *P* value of <0.003 as the significance threshold.

To assess whether the differences between the 2005 to 2010 and 2011 to 2015 groups would lead to a difference in study outcomes, the association of age with perioperative outcomes was assessed as an example analysis. Multivariate Poisson regression was conducted to study the incidence of postoperative adverse outcomes with increasing age. A Bonferroni correction was again used to correct for the multiple tests carried out, yielding a *P* value of <0.005 as the significance threshold.

## Results

### Total Hip Arthroplasty Patient Populations

A total of 102,411 patients were identified from 2005 to 2015 in the NSQIP using the aforementioned inclusion and exclusion criteria. Of these, 8098 cases were from the 2005 to 2010 data set and 94,313 were from the 2011 to 2015 data set.

Table 1 presents demographic data for the total study population and the two era subcohorts, along with bivariate comparisons for each of the variables between the two era groups. Race, alcohol use, ASA class, diabetes, hypertension, cardiac comorbidity, neurologic comorbidity, and functional status were all significantly different between the two groups (*P* < 0.003). Fewer white and Hispanic patients were included in the 2011 to 2015 cohort versus the 2005 to 2010 group. The proportion of functionally independent patients increased from 2005 to 2010 to 2011 to 2015. Conversely, the average ASA class and the prevalence of alcohol use, hypertension, cardiac comorbidity, and neurologic comorbidity decreased from older to more recent years.

**Table 1:**
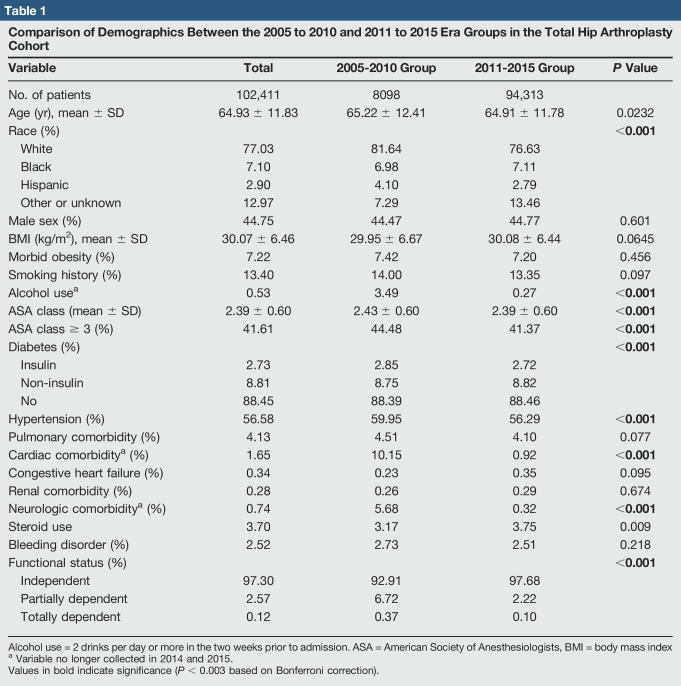
Comparison of Demographics Between the 2005 to 2010 and 2011 to 2015 Era Groups in the Total Hip Arthroplasty Cohort

Variable	Total	2005-2010 Group	2011-2015 Group	*P* Value
No. of patients	102,411	8098	94,313	
Age (yr), mean ± SD	64.93 ± 11.83	65.22 ± 12.41	64.91 ± 11.78	0.0232
Race (%)				**<0.001**
White	77.03	81.64	76.63	
Black	7.10	6.98	7.11	
Hispanic	2.90	4.10	2.79	
Other or unknown	12.97	7.29	13.46	
Male sex (%)	44.75	44.47	44.77	0.601
BMI (kg/m^2^), mean ± SD	30.07 ± 6.46	29.95 ± 6.67	30.08 ± 6.44	0.0645
Morbid obesity (%)	7.22	7.42	7.20	0.456
Smoking history (%)	13.40	14.00	13.35	0.097
Alcohol use^a^	0.53	3.49	0.27	**<0.001**
ASA class (mean ± SD)	2.39 ± 0.60	2.43 ± 0.60	2.39 ± 0.60	**<0.001**
ASA class ≥ 3 (%)	41.61	44.48	41.37	**<0.001**
Diabetes (%)				**<0.001**
Insulin	2.73	2.85	2.72	
Non-insulin	8.81	8.75	8.82	
No	88.45	88.39	88.46	
Hypertension (%)	56.58	59.95	56.29	**<0.001**
Pulmonary comorbidity (%)	4.13	4.51	4.10	0.077
Cardiac comorbidity^a^ (%)	1.65	10.15	0.92	**<0.001**
Congestive heart failure (%)	0.34	0.23	0.35	0.095
Renal comorbidity (%)	0.28	0.26	0.29	0.674
Neurologic comorbidity^a^ (%)	0.74	5.68	0.32	**<0.001**
Steroid use	3.70	3.17	3.75	0.009
Bleeding disorder (%)	2.52	2.73	2.51	0.218
Functional status (%)				**<0.001**
Independent	97.30	92.91	97.68	
Partially dependent	2.57	6.72	2.22	
Totally dependent	0.12	0.37	0.10	

Alcohol use = 2 drinks per day or more in the two weeks prior to admission. ASA = American Society of Anesthesiologists, BMI = body mass index

a Variable no longer collected in 2014 and 2015.

Values in bold indicate significance (*P* < 0.003 based on Bonferroni correction).

Average surgical time is reported for the two cohorts in Table 2, along with bivariate comparison. Surgical time was mildly but significantly lower in the 2011 to 2015 cohort versus 2005 to 2010 (*P* < 0.05).

**Table 2:**
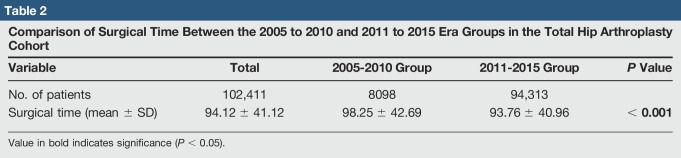
Comparison of Surgical Time Between the 2005 to 2010 and 2011 to 2015 Era Groups in the Total Hip Arthroplasty Cohort

Variable	Total	2005-2010 Group	2011-2015 Group	*P* Value
No. of patients	102,411	8098	94,313	
Surgical time (mean ± SD)	94.12 ± 41.12	98.25 ± 42.69	93.76 ± 40.96	**< 0.001**

Value in bold indicates significance (*P* < 0.05).

Subsequent postoperative adverse outcomes are shown in Table 3 with bivariate comparisons. The prevalence of urinary tract infection (UTI), blood transfusion, and sepsis, and the average LOS and extended LOS were significantly different between the two groups (*P* < 0.003). There were significantly fewer UTIs, cases of sepsis, and extended lengths of stay in 2011 to 2015 versus 2005 to 2010. The incidence of blood transfusion increased significantly between the two groups. The average LOS decreased significantly between the two periods as well.

**Table 3:**
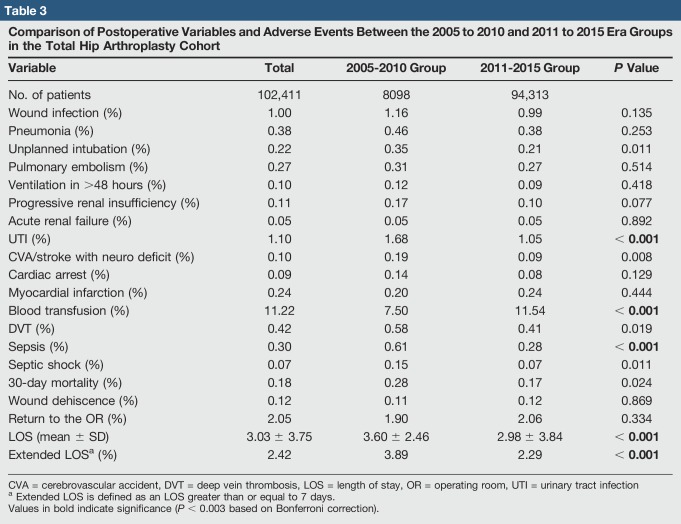
Comparison of Postoperative Variables and Adverse Events Between the 2005 to 2010 and 2011 to 2015 Era Groups in the Total Hip Arthroplasty Cohort

Variable	Total	2005-2010 Group	2011-2015 Group	*P* Value
No. of patients	102,411	8098	94,313	
Wound infection (%)	1.00	1.16	0.99	0.135
Pneumonia (%)	0.38	0.46	0.38	0.253
Unplanned intubation (%)	0.22	0.35	0.21	0.011
Pulmonary embolism (%)	0.27	0.31	0.27	0.514
Ventilation in >48 hours (%)	0.10	0.12	0.09	0.418
Progressive renal insufficiency (%)	0.11	0.17	0.10	0.077
Acute renal failure (%)	0.05	0.05	0.05	0.892
UTI (%)	1.10	1.68	1.05	**< 0.001**
CVA/stroke with neuro deficit (%)	0.10	0.19	0.09	0.008
Cardiac arrest (%)	0.09	0.14	0.08	0.129
Myocardial infarction (%)	0.24	0.20	0.24	0.444
Blood transfusion (%)	11.22	7.50	11.54	**< 0.001**
DVT (%)	0.42	0.58	0.41	0.019
Sepsis (%)	0.30	0.61	0.28	**< 0.001**
Septic shock (%)	0.07	0.15	0.07	0.011
30-day mortality (%)	0.18	0.28	0.17	0.024
Wound dehiscence (%)	0.12	0.11	0.12	0.869
Return to the OR (%)	2.05	1.90	2.06	0.334
LOS (mean ± SD)	3.03 ± 3.75	3.60 ± 2.46	2.98 ± 3.84	**< 0.001**
Extended LOS^a^ (%)	2.42	3.89	2.29	**< 0.001**

CVA = cerebrovascular accident, DVT = deep vein thrombosis, LOS = length of stay, OR = operating room, UTI = urinary tract infection

a Extended LOS is defined as an LOS greater than or equal to 7 days.

Values in bold indicate significance (*P* < 0.003 based on Bonferroni correction).

### Example Analysis of the Correlation of Age With Perioperative Outcomes

**Figure 1:**
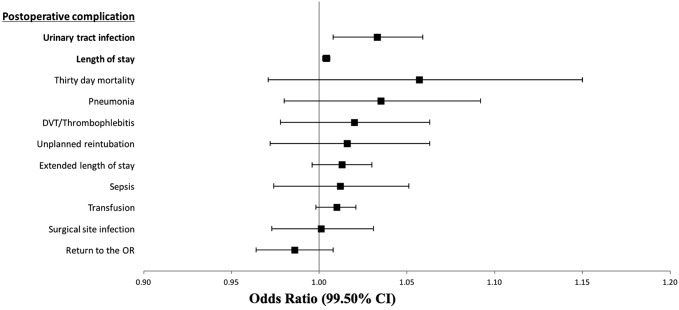
Illustration of the association of increasing age with perioperative complications from 2005 to 2010 (n = 8098). Relative risk is for age as a continuous variable. CI = confidence interval, DVT = deep vein thrombosis, OR = operating room

**Figure 2:**
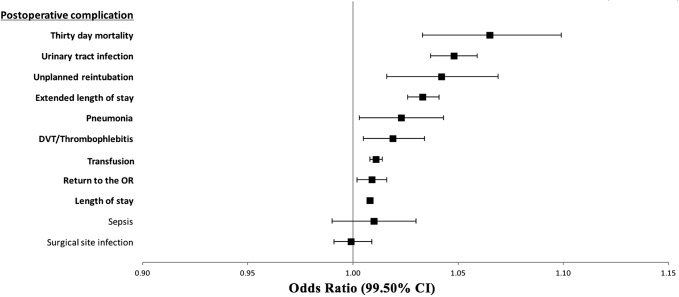
Illustration of the association of increasing age with perioperative complications from 2011 to 2015 (n = 94,313). Relative risk is for age as a continuous variable. CI = confidence interval, DVT = deep vein thrombosis, OR = operating room

Finally, the association between increasing age and postoperative events was assessed for the two study groups. The results of these analyses are shown in Table 4 and Figures 1 and 2.

**Table 4:**
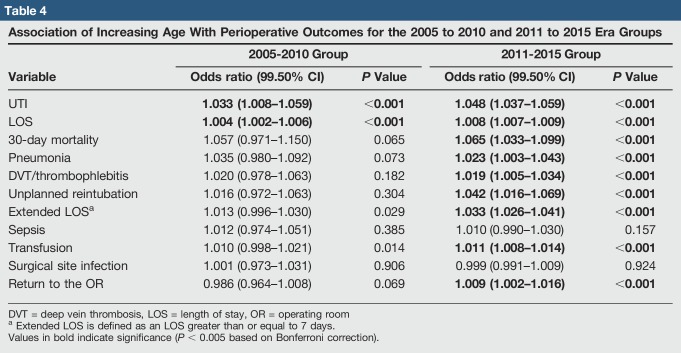
Association of Increasing Age With Perioperative Outcomes for the 2005 to 2010 and 2011 to 2015 Era Groups

Variable	2005-2010 Group		2011-2015 Group	
	Odds ratio (99.50% CI)	*P* Value	Odds ratio (99.50% CI)	*P* Value
UTI	**1.033 (1.008–1.059)**	**<0.001**	**1.048 (1.037–1.059)**	**<0.001**
LOS	**1.004 (1.002–1.006)**	**<0.001**	**1.008 (1.007–1.009)**	**<0.001**
30-day mortality	1.057 (0.971–1.150)	0.065	**1.065 (1.033–1.099)**	**<0.001**
Pneumonia	1.035 (0.980–1.092)	0.073	**1.023 (1.003–1.043)**	**<0.001**
DVT/thrombophlebitis	1.020 (0.978–1.063)	0.182	**1.019 (1.005–1.034)**	**<0.001**
Unplanned reintubation	1.016 (0.972–1.063)	0.304	**1.042 (1.016–1.069)**	**<0.001**
Extended LOS^a^	1.013 (0.996–1.030)	0.029	**1.033 (1.026–1.041)**	**<0.001**
Sepsis	1.012 (0.974–1.051)	0.385	1.010 (0.990–1.030)	0.157
Transfusion	1.010 (0.998–1.021)	0.014	**1.011 (1.008–1.014)**	**<0.001**
Surgical site infection	1.001 (0.973–1.031)	0.906	0.999 (0.991–1.009)	0.924
Return to the OR	0.986 (0.964–1.008)	0.069	**1.009 (1.002–1.016)**	**<0.001**

DVT = deep vein thrombosis, LOS = length of stay, OR = operating room

a Extended LOS is defined as an LOS greater than or equal to 7 days.

Values in bold indicate significance (*P* < 0.005 based on Bonferroni correction).

For the 2005 to 2010 cohort (Table 4 and Figure 1), UTI and LOS were the only two postoperative complications found to be significantly associated with increased age. For the 2011 to 2015 cohort (Table 4 and Figure 2), age was found to be significantly associated with 30-day mortality, LOS, return to the operating room (OR), the incidence of UTI, unplanned reintubation, extended LOS, pneumonia, deep vein thrombosis/thrombophlebitis, and transfusion.

## Discussion

National databases facilitate outcomes studies by providing large cohorts of patients collected from many institutions over multiple years. The American College of Surgeons' NSQIP database is commonly used for research, as it provides rigorously collected data and tracks patients for 30 days postoperatively. However, the database has changed over the years with regard to its patient population and variable collection practices. Few studies have addressed how the systemic changes in the NSQIP over the years may influence the study results.

One previous study, which showed that using only older lumbar fusion NSQIP data (before 2011) resulted in different outcomes than more recent NSQIP data, raised concern that older NSQIP studies might not fully characterize the current outcomes.^25^ Directly related to this for THA, multiple studies published in the past 4 years looking at THA have used only data obtained before 2011.^18,19,20,21,22,23,24^ For this reason, the current study sought to characterize changes within the NSQIP between 2005 to 2010 and 2011 to 2015 for THA patients and any potential effect on study outcomes. In fact, these NSQIP THA subcohorts were found to be associated with differences in preoperative characteristics and perioperative outcomes which led to different results between the era groups for an example analysis of the association of age with perioperative outcomes.

In terms of the sample size, the number of patients included in the NSQIP has increased dramatically between older and newer years. The 2005 to 2010 cohort of THA patients yielded 8098 patients, whereas the 2011 to 2015 cohort contained 94,313 patients. The number of participating sites these patients were drawn from increased from 121 to 603 over the same interval. Concurrently with this growth, changes in patient characteristics over the years were also noted, as shown by the data presented in the current study.

One potential reason for the changing patient characteristics seen over the database years is the preoperative evaluation of those undergoing THA between the 2005 to 2010 and 2011 to 2015 groups. The increase in functionally independent patients undergoing THA may be consistent with THA being performed earlier in the disease course. Studies have shown that patients with better preoperative function and lower comorbidity burden may see improvements in postoperative outcomes.^24,25,26,27,28^ Similarly, the decrease in ASA and hypertension may be consistent with THA being performed in more medically optimized patients. The mild but significant decrease in surgical time that was seen between the two groups may be consistent with improving surgical techniques.

By contrast, other changes over the database years may be more likely related to changes in variable collection and definitions. For example, the incidence of transfusion increased roughly 4% between 2005 to 2010 and 2011 to 2015 despite the fact that the trigger for transfusion has generally become more stringent over the years. Before 2011, the NSQIP indicated transfusion if blood was given in the first 72 hours postoperatively or for blood hung in the OR but finished outside of it if greater than 5 units were given. After 2011, the NSQIP started to include blood given at any time after the start of surgery, for any amount given.

With the changes in patient population and data collection over the years in mind, there is the question of how much of an impact this could have on THA studies based on data from newer years versus older years. To that end, an example analysis of the effect of patient age on study outcomes was performed and clear differences were observed. Multivariate analysis of the 2005 to 2010 group showed that increasing age was only associated with UTI and LOS, whereas multivariate analysis of the 2011 to 2015 group showed that age was not only significantly associated with UTI and LOS but also with 30-day mortality, unplanned reintubation, extended LOS, pneumonia, deep vein thrombosis/thrombophlebitis, transfusion, and return to the OR. As discussed earlier, at least eight studies from the last 4 years used only data before 2011.^11,18,19,20,21,22,23,24^ It is possible that these studies' analyses would yield different results if repeated using newer data, as was the case in the example analysis given previously.

One explanation for the increase in significant findings for the earlier multivariate analysis is a simple increase in power between the two sets of data years, without a corresponding definitional change. As an example of this, the odds ratio for the association of increased patient age with pneumonia decreased between 2005 to 2010 and 2011 to 2015 but went from a nonsignificant to a significant association with the markedly increased power of the analysis. However, another explanation for some of the changes in association could also be related to the changes in data elements and definitions discussed earlier. These changes, and the fact that so many more patients are present in the later years of the NSQIP data set, should make one question using only the earlier years of NSQIP data in longitudinal studies.

The current study does have limitations. First, the current analysis is specific to the NSQIP database. That said, this is a commonly used database in the field, as can be seen by the many recent studies cited.^1,2,3,4,5,18,19,20,21,22,23,24^ Second, it is possible that the observations made here may not apply to other NSQIP study populations. That said, the overall conclusions made here mirror those of the previously referenced lumbar study.^25^

## Conclusions

National databases such as the NSQIP continue to be a common source of data for studies of outcomes after THA. Despite the ability to create large cohorts of patients spanning multiple years, the utility of the NSQIP is limited by systemic changes in population composition and variable collection practices throughout the years. As shown in this study, these changes may contribute to different results for identical analyses when using older versus newer data. The results of this study suggest that studies done with data from only years before 2011 may need to be reconsidered (Supplemental Digital Content 1, http://links.lww.com/JG9/A58; and Supplemental Digital Content 2, http://links.lww.com/JG9/A59).

## Supplementary Material

SUPPLEMENTARY MATERIAL
